# Typologies of Adolescent Musicians and Experiences of Performance Anxiety Among Instrumental Learners

**DOI:** 10.3389/fpsyg.2021.645993

**Published:** 2021-05-06

**Authors:** Ioulia Papageorgi

**Affiliations:** Department of Social Sciences, Faculty of Humanities and Social Sciences, University of Nicosia, Nicosia, Cyprus

**Keywords:** music performance anxiety, adolescence, typology, K-means cluster analysis, thematic analysis

## Abstract

Literature suggests that music performance anxiety (MPA) is prevalent in adolescence, a developmental period with increased likelihood of experiencing anxiety under evaluative conditions. Evidence also indicate that individuals may respond to evaluative situations in distinct ways. Factors contributing to the individuality of responses in evaluative situations (such as test taking and musical performance) are not yet fully understood. This study investigated student typologies in adolescent instrumental learners. Participants included 410 learners who completed the Young Musicians’ Performance Questionnaire. K-Means cluster analysis revealed three typologies: Cluster 1 – moderately anxious students evidencing lower levels of motivation and feeling ineffective but guarding their self-esteem; Cluster 2 – highly anxious students evidencing negative self-perceptions and being susceptible to experiencing maladaptive MPA; Cluster 3 – low anxious students evidencing high levels of motivation and confidence and inclined toward experiencing adaptive MPA. The 3-cluster solution effectiveness was validated with discriminant analysis. Significant associations between examination achievement and cluster membership revealed variations between clusters. Thematic analysis of qualitative data facilitated further understanding of their characteristics. This study adds to the body of MPA literature by exploring the different ways with which adolescent musicians interpret and respond to anxiety inducing situations. Findings have implications for clinical and educational practice.

## Introduction

Musical performance engages auditory and visual perception skills, attention, precise timing, extended control over movement, learning, memory, and emotion (Kanduri et al., 2015). Developing expertise in instrumental performance is a complex process requiring the development of aural, cognitive, technical, musical, communication, and performing skills (Hallam, 2006), and is categorised under complex skill learning. Music performance is an activity that is physically and psychologically demanding (Papageorgi and Kopiez, 2018) and can thus place physical and psychological stress on performers (Vervainioti and Alexopoulos, 2015). One of the most common psychological stressors reported in the literature is music performance anxiety (MPA) (Barbar et al., 2014; Kenny et al., 2014; Burin and Osorio, 2017; Fernholz et al., 2019). Symptoms can be categorised under physiological (changes in the physiological state of the organism), mental (cognitive and emotional) and behavioural (visible changes in behaviour) and are experienced concurrently (Burin and Osorio, 2017). A major concern for musicians is that MPA can bring about practical complications and negatively affect the quality of performance, irrespective of the level of preparation and ability of the performer (Papageorgi and Welch, 2014a). MPA can vary in severity, to the extent of even forcing some musicians who are highly affected to put an end in their careers (Fernholz et al., 2019).

Contemporary conceptualisations of MPA view it as a multidimensional construct (Wilson, 2002; Osborne and Kenny, 2005; Papageorgi et al., 2007; Kenny et al., 2014). Contemporary theoretical models take into account the complexity of individual, task-related, and situational variables in the development and manifestation of MPA and view its development as a process that unfolds in a time sequence: pre-, during-, and post- performance (Papageorgi et al., 2007).

### Prevalence of MPA

Music performance anxiety prevalence in the literature has been mostly assessed through self-report psychometric tests and not by professionals on the basis of ICD or DSM criteria (Fernholz et al., 2019). Evidence suggests that MPA is highly prevalent in musicians (both children/adolescents and adults), but there is no consensus on prevalence rates. Between 15 and 70% of adult orchestral and choral musicians report the experience of MPA (Schulz, 1981; Gustafson and Rawson, 1983; Fishbein et al., 1988; Marchant-Haycox and Wilson, 1992; Steptoe, 2001; Brugués, 2009; Ryan and Andrews, 2009; Medeiros Barbar et al., 2014). A recent systematic review of MPA in professional musicians reported prevalence rates between 16.5 and 60% (Fernholz et al., 2019), noting that when focusing on reports indicating MPA to be a severe problem for musicians the evidence suggest that approximately 1/3 of musicians suffer from MPA.

Student musicians also report the presence of MPA to a significant extent. Wesner et al. (1990) reported that 21% of their sample of university students suffered from severe distress and 40% experienced moderate distress due to performance anxiety. American music students reported performance anxiety as one of the most disturbing and worrying issues in their lives (Dews and Williams, 1989). Around 50% of Swedish student and professional performers stated that they had often encountered performance anxiety, and 10% said that they always felt high distress before performing (Gustafson and Rawson, 1983). MPA was one of the most frequently reported problems for conservatoire students in the United Kingdom (Williamon and Thompson, 2006).

In younger musicians, Papageorgi (2020) reported that in a sample of 410 adolescent musicians, 11% reported high MPA levels. Fehm and Schmidt (2006) found that 33% of adolescent musicians experienced a negative impact of MPA, and about 10% reported that their musical career was negatively affected.

### MPA in Adolescence

Adolescence has been reported as a developmental period with increased vulnerability to MPA (Fehm and Schmidt, 2006; Kenny and Osborne, 2006). Younger musicians’ experiences of MPA in terms of physiological, cognitive and behavioural symptoms are similar to those of adult musicians (Ryan, 1998, 2004; Fehm and Schmidt, 2006; Osborne and Kenny, 2008). Anxiety can also be evident in the early years. Boucher and Ryan (2011), using self-report, physiological and observational data found that children as young as 3–4 years old experience MPA. Recently, Zarza-Alzugaray et al. (2016) found that onset of musical training at or before the age of 7 may act as a protective barrier against MPA in music school pupils (mean age 12 years old).

A small number of cohort studies looked at the developmental trajectory of MPA in adolescence. Evidence suggest that that MPA increases with age as older adolescents appear to be more anxious (Osborne et al., 2005; Patston and Osborne, 2016). Dempsey and Comeau (2019) compared MPA in child (aged 7–12) and adolescent musicians (aged 13–17) and found that MPA levels increased with age, with adolescents experiencing significantly higher anxiety levels.

Papageorgi (2020) found that MPA in adolescents increased between the ages of 15 and 18, and decreased at the age of 19. The study also found evidence of cultural- and sex-specific variations in the manifestation of MPA. Girls experienced higher levels of MPA compared to boys, in agreement with the trend reported in the literature (Rae and McCambridge, 2004; Kenny and Osborne, 2006; Papageorgi, 2008; Papageorgi et al., 2013; Thomas and Nettelbeck, 2014; Patston and Osborne, 2016). In terms of the developmental trajectory, there was a steep increase in girls’ levels of MPA between the ages of 15 and 18, a finding corroborating Patston and Osborne (2016) who also found that females experienced a steeper and more intense developmental trajectory.

The developmental trajectory may be affected by factors such as the personality and chosen musical genre of the performer, which may yield differentiations. Sarbescu and Dorgo (2014) in their study of 14–19 years old musicians reported that it was younger students who also had lower levels of emotional stability and lower performance frequency who reported higher levels of MPA. A further issue that must be taken into account is that the developmental trajectory of MPA may depend on the musical genre, with different developmental MPA profiles for classical and popular musicians. Nusseck et al. (2015), in a study of 7–20 years old music school students found that younger classical musicians (under age 16) were more anxious compared to their older counterparts, whereas the opposite trend was observed in popular musicians with older musicians (age 16+) evidencing higher MPA levels.

### Individual Differences in Responses to Performance Anxiety

Individuals do not respond to evaluative situations in the same way. Evidence suggests that individuals with different characteristics (e.g., age, gender, experience, and expertise level) can perceive and respond to conditions giving rise to performance anxiety or test anxiety in distinct ways (Losiak, 2005; Chow and Mercado, 2020).

Chow and Mercado (2020) looked at how task-specific expertise and past experiences moderate the degree to which individuals become anxious in a given performance context. Authors considered how individual differences arising from learning can influence the psychobiological, emotional, and cognitive processes that modulate anxious states during the performance of highly trained skills (such as in musical performance and sport performance). Their review concluded that experience dependent brain plasticity determines how a person will respond to each unique performance context. Authors suggested that in order to identify the processes that lead to maladaptive (problematic) or adaptive (advantageous) performance anxiety, theories of performance anxiety that clarify for how past experiences shape the emergence of emotional states that trigger cognitive appraisals are needed.

Nielsen et al. (2018) investigated the relationship between MPA, subjective performance quality and post-event rumination (PER) in university music students. Authors found that high-anxious music students showed more negative and less positive PER compared to the low-anxious music students after a 10-min solo performance. Furthermore, the development pattern for negative PER was related to MPA level. High-anxious musicians evidenced a slower decrease of negative PER compared to low- and moderate-anxious performers. These results suggest that the quality of musicians’ responses to anxiety-inducing performances is dependent upon their MPA level, with those susceptible to higher MPA being more likely to need more time to recover after a performance.

### Profiles of Test Takers and Test/Performance Anxiety

The recognition that individuals can respond to anxiety-inducing evaluative situations in different ways has steered research to investigate the presence of profiles or types of test takers in relation to anxiety.

#### Test Anxiety Literature

Cassady and Finch (2015) undertook a study with undergraduate students to explore whether it is possible to identify subcomponents of the cognitive test anxiety construct, hypothesising the presence of “types” of test anxious learners. They observed differences in the way low and high anxious individuals conceptualised cognitive test anxiety. Those with low levels of cognitive test anxiety understood cognitive test anxiety as a unidimensional construct. High test anxious students, however, represented it as a more complex construct and differentiated between two different aspects: Cognitive interference (both during the preparation stage and the performance phase) and perceived skill deficiencies in test taking. Their results suggest that the level of anxiety experienced is associated with how cognitive test anxiety is understood by learners.

Davis et al. (2008) employed cluster analysis in a sample of university students, exploring patterns of appraising tests. Authors were able to identify five subtypes (clusters) of test takers who varied their approach toward tests, their experience of anxiety, as well as how they coped with problems occurring during test taking: (1) Tests out of control; (2) Well-prepared for challenges; (3) Feeling hopeless; (4) Keeping tests in perspective; and (5) Bracing for the worst. These findings indicate that different profiles of test takers exist, differentiated by their overall approach toward tests, their experience of anxiety, and coping patterns utilised to manage test anxiety.

Putwain and Daly (2013) investigated whether secondary school students can be distinguished in distinct clusters on the basis of their test anxiety and academic buoyancy (resilience) scores, as well as their academic performance. Cluster analysis revealed the presence of five clusters with diverse combinations of test anxiety level and academic resilience. Cluster 1 represented high anxiety/low academic buoyancy and had the lowest academic performance. Cluster 2 corresponded to low anxiety/high academic buoyancy and had the highest academic performance. Cluster 3 represented moderate anxiety/moderate academic buoyancy. Cluster 4 represented moderate-high test anxiety/moderate buoyancy. Cluster 5 represented moderate anxiety/high buoyancy. These results highlight the distinctiveness of student responses to performance evaluative situations. Differences in academic performance between the clusters was also noted. Higher performance was observed in students exhibiting high academic buoyancy and either low (suggestive of lower threat appraisal) or moderate (signifying a buffering effect on performance) test anxiety levels.

In another study, Losiak (2005) investigated anxiety profiles in a non-clinical sample of undergraduate students. Cluster analysis suggested the presence of six clusters, which were categorised into two groups of profiles. The first group of profiles included three clusters in which the participants had average, moderate and low anxiety levels. These profiles were not differentiated, as no form of anxiety reactions or anxiety provoking situations dominated – they were rather unitary in characteristics and did not score extremely in anxiety measures. The second group of profiles included three clusters that were quite differentiated. One cluster included extremely anxious individuals who experienced motor anxiety in phobic situations. Another cluster included high anxious individuals characterized by physiological anxiety presented in daily life and evaluative situations (e.g., in exams, public speaking). The third cluster included participants who were very low anxious with a tendency to experience physiological anxiety in phobic and interpersonal situations (e.g., a date or social meetings). This study suggests that individuals can respond to anxiety-inducing situations in distinct ways.

Mammarella et al. (2018) conducted a study with primary school children to investigate the presence of subgroups expressing particular anxiety patterns on the basis of measures of general anxiety (GA), test anxiety (TA), and maths anxiety (MA). Authors also explored the role of personal factors protecting against anxiety (such as self-concept, resilience, academic self-concept, and academic buoyancy). Three clusters of students were identified: Low risk students (low scores in various forms of anxiety); Average risk students (average scores in anxiety measures); and High-risk students (high scores in anxiety measures) anxiety. The three clusters differed in their experiences of the different types of anxiety. For example, whereas the low risk group had low scores in all measures (GA, TA, and MA), the average risk students scored higher in GA and MA, and lower in TA. The high risk students evidenced higher levels of GA and TA, but lower levels of MA. Children with a low-risk profile reported a more positive academic self-concept and felt more competent overall compared to the other two groups.

Carey et al. (2017) measured math anxiety, test anxiety, general anxiety and mathematics and reading performance in primary school children in the United Kingdom. Latent Profile Analysis revealed different anxiety “profiles” in year 4 students and year 7 and 8 students. Year 4 students’ data analysis produced four profiles: Low anxiety, Slight anxiety, Moderate anxiety and High anxiety. Scores in the various anxiety measures were homogenous within each profile. The analysis of year 7 and 8 students’ scores revealed four groups with varying patterns of scores on each of the three anxiety measures. One group included participants with normative scores on all measures (Low anxiety). The second group evidenced high scores only on the measure of general anxiety (General anxiety). The third group scored highly on test anxiety and mathematics anxiety (Academic anxiety). The fourth group had high scores on all three anxiety measures (High anxiety). This study provides further evidence for the presence of differences in anxiety responses between students, and also suggests that there may be a developmental trajectory.

Stenlund et al. (2018) conducted a study aiming to identify and group test takers with similar patterns of test-taking behaviour and to investigate how these groups differ in relation to background characteristics and test performance in a high-stakes achievement test context (the Swedish SAT). Cluster analysis revealed three clusters of test takers with significantly different test-taking behaviour profiles: Moderate (*n* = 741), Calm Risk Taker (*n* = 637), and Test Anxious Risk Averse (*n* = 513). The calm risk taker profile (high level of risk taking, low levels of test anxiety and motivation) had the higher performance. The test anxious risk averse profile (low degree of risk taking, high levels of test anxiety and motivation) had the lower performance.

In a recent study, Wang et al. (2020) looked at the developmental trajectory of mathematics anxiety (MA) in secondary school students and its association with cognitive, personality and environmental factors. Results identified four distinct growth trajectories of maths anxiety: (1) A non-anxious group (exhibited chronically low MA); (2) A highly anxious group (displayed moderately high MA over time); (3) A resilient group (exhibited high initial MA but it steadily decreased over time); and (4) A vulnerable group (reported low initial MA but it drastically increased over time). Researchers pointed that their findings highlight the presence of heterogeneity in the development of MA, and identified middle school as a critical period for MA development. They also called attention to the importance of examining developmental changes in cognitive, personality, and environmental factors in elucidating distinct MA trajectories in secondary school.

#### MPA Literature

Wiedemann et al. (2019) investigated MPA and its anxiety correlates in undergraduate music students. They were able to identify, through cluster analysis, two groups of participants evidencing different patterns of anxiety. Cluster 2 showed elevated anxiety levels in nearly all DSM-5 anxiety measures used (e.g., generalised anxiety disorder and social anxiety disorder). On the contrary, Cluster 1 did not display pathological anxiety symptoms in the DSM-5 anxiety measures, but their MPA included both lower and higher levels. Findings support the differentiation between different MPA types. These may include MPA as an important disorder presented in an otherwise healthy musician, and MPA as part of a more complex psychopathology.

### The Current Study

The review of the literature suggests that MPA is prevalent in adolescence. Test anxiety literature also suggests that individuals may perceive and respond to evaluative situations in distinct ways, resulting in the presence of distinct profiles of test takers in relation to the experience of test anxiety. The factors that contribute to the individuality of responses in evaluative situations (such as test taking and musical performance) are not yet fully understood. Test anxiety literature has made progress in this area, by investigating through cluster analysis, the presence of profiles of test takers in relation to their test anxiety levels and achievement. With the exception of Wiedemann et al. (2019), MPA literature has not addressed this issue and so there is limited knowledge on the presence of typologies of instrumental learners in relation to performance anxiety.

The aim of the current study was to investigate the presence of student typologies in adolescent learners and explore associations with experiences of MPA and achievement in instrumental examinations. To the best knowledge of the author, no studies have investigated student typologies and MPA in adolescent learners and, as such, the current study has the potential to make a novel contribution to the literature. The current study focuses on classical musicians, who in the literature have been reported as more likely to experience higher MPA levels (Papageorgi et al., 2013; Papageorgi and Welch, 2014b; Perdomo-Guevara, 2014; Nusseck et al., 2015).

Findings from relevant studies in the field of test anxiety have guided the development of the research questions of the study. Davis et al. (2008) found different profiles of students based on test anxiety experienced and coping strategies they used. More recently, Putwain and Daly (2013) found different profiles of test takers with each profile evidencing different achievement levels. The investigation sought to explore whether similar phenomena occur with instrumental students and MPA. Literature on MPA has not yet explored whether distinguishable typologies/profiles of musicians can be detected on the basis of correlates of MPA and, in such case, whether different typologies are associated with different levels of achievement in instrumental examinations. To explore these issues, the following research questions were devised:

1. Is it possible, through cluster analysis, to identify distinct profiles (typologies) of adolescent musicians, based on correlates of performance anxiety and experiences of MPA?
2. If typologies of adolescent musicians are identified, how do the clusters relate to achievement in instrumental examinations?

## Materials and Methods

### Participants

Four hundred and ten adolescent musicians took part in the study (ages 12–19 *M*_age_ = 15.33). Data were collected in two geographical locations, Cyprus (51.5% of participants) and the United Kingdom (48.5% of participants). The decision to collect data from two geographical locations was driven by one of the main aims of the overall study, which was to investigate cultural variability in MPA, with relevant findings reported in an earlier publication (Papageorgi, 2020). All participants attended junior conservatoires where they undertook instrumental lessons, and/or played in youth orchestras. There were slightly more females (58%) compared to males (42%). Participants played a range of instruments, including piano/keyboard, string, woodwind, brass, percussion instruments, guitar, voice, and harp and identified themselves as classical musicians.

As participants came from two different geographical locations and nationalities, a comparison of the two nationality samples was initially conducted to assess their homogeneity and ascertain whether data should be analysed separately or collectively. Chi-square test analyses assessing the association between nationality and basic demographics of sex (males and females) and age group (younger adolescents 12–15, older adolescents 16–19) revealed no significant differences (sex *p* = 0.554; age group *p* = 0.711). Some statistically significant differences were observed in years playing first instrument and grade level, indicating that more British students played for longer [8+ years; *x*^2^(5) = 46.55, *p* < 0.001] and were placed at advanced grades [grade 8; *x*^2^(8) = 75.20, *p* < 0.001]. There was no significant association between the two nationalities in attainment as measured by the assessment grade received in the last exam taken (*p* = 0.289). Overall, the results suggested that the Cypriot and British samples were homogenous in basic characteristics and attainment, although the British students were slightly more advanced. Taking the evidence into consideration, it was decided to analyse data collectively.

### Materials

The Young Musicians’ Performance Questionnaire (Papageorgi, 2007a, 2020) was used to collect data pertaining to learning, performance and experiences of musical performance anxiety, as part of a study investigating performance anxiety in adolescent musicians. Students completed the questionnaire on paper during a break from rehearsals or class. The items comprising the questionnaire were validated by two experts in the field during the development phase and prior to a pilot study (for further details, please see Papageorgi, 2020). Following necessary modifications after the pilot study, the questionnaire was finalised and then translated in Greek for the Cypriot participants using backward translation (for further details, see Papageorgi, 2020).

The questionnaire was broken down into four sections. Section A requested demographic information [sex, age, nationality, instrument(s) played, years playing, grade level, and mark (assessment grade) obtained in the last examination taken]. Section B included 54 statements (items) to which participants were requested to indicate their degree of agreement on a five-point Likert-type scale (1 = strongly disagree; 5 = strongly agree). The items comprised potential MPA predictor variables or correlates, identified on the basis of previous literature, with a focus on the three thematic areas suggested by Papageorgi et al. (2007) and Burin and Osorio (2017): the performer, the task and perceptions of the environment. Each of these variables was explored via items capturing its characteristics and related behaviours, as reported in the literature. They were grouped into 18 variables/mini scales (three items per variable/short scale), recoding any items necessary to ensure agreement in conceptual direction. Hair et al. (2010, p. 669) suggested that three items can ensure adequate coverage of a construct’s theoretical domain. Furthermore, Gogol et al. (2014) suggested that short forms (three-item or single item) of domain-general and domain-specific academic anxiety measures can be recommended as psychometrically sound alternatives when study designs require brief measures. Internal reliability analyses of the short scales comprising each variable indicated acceptable internal consistency, with all Cronbach’s α values >0.6. For the purposes of the current article, the variables indicated in Table 1 were used (for further details see Papageorgi, 2020).

**TABLE 1:** Variables included in section B of the questionnaire.

Variable	Example item
Entity theory of ability	Talent is the most important component of success for a performer
Fear of evaluation	If I do poorly in a performing situation, others will question my ability in music
Incremental theory of ability	With lots of practice, one can accomplish anything.
Perception of critical parents with high expectations	The comments of my family about my performances are critical
Perception of supportive and encouraging parents	My parents are always supportive
Perception of receiving positive feedback from teacher	My teacher believes I could go on to be a professional musician
Perception of being under pressure to continue with music lessons	I am worried about how my parents would react if I decided to stop going to music lessons
Development of musical identity	My dream is to become a professional performer
Negative perception of anxiety	Anxiety can destroy the career of a musician
Positive self-concept in music	When I compare myself with other young performers I think that I am very good
Perfectionism	I am sad if I do not get a good result in an exam/audition
Effortful practice	I usually practice very hard
Low self-efficacy in music	I avoid working on pieces of music that look or sound difficult
Intrinsic motivation to learn a musical instrument	I enjoy learning to play a musical instrument and I want to be good at it
Experience of heightened anxiety in the presence of an audience	I am more nervous when I know someone is listening to me playing
Sensitivity to degree of self-exposure	Solo performances are scarier than group performances
Sensitivity to environmental conditions	I find that when I do not feel comfortable in the venue where I have to play, I am more anxious than usual

Section C included twenty items that together formed the Adolescent Musicians’ Performance Anxiety Scale (AMPAS) (see Papageorgi, 2007a, b, 2020). Participants were asked to indicate the frequency of experiencing particular symptoms and cognitions on a five-point Likert type scale (1 = never; 5 = always). The possible range of scores ranged from 20 to 100, with higher scores indicating higher performance anxiety levels. The items were organised in subscales or variables/themes that reflected the multidimensional nature of MPA as exemplified in previous literature and, for the purposes of the current analysis, were combined into a new variable using a similar procedure as in Section B: Negative outcome expectancies (two items), negative experiences in performance (five items), evidence of pre-evaluation anxiety (three items), experience of physiological symptoms of anxiety (four items), concern about others’ judgement (two items) and experience negative effects of anxiety (four items). Assessment of the psychometric properties of the AMPAS scale suggests that it is a promising psychometric instrument to measure performance anxiety in this population, with good internal reliability (Cronbach’s α = 0.86) and 2 week test-retest reliability (*r* = 0.91), and good construct validity (*r* = 0.74 with KMPAI; Kenny, 2009 and *r* = 0.80 with PAI, Nagel and Himle, 1989) (for further details see Papageorgi, 2020). Factor analysis revealed the presence of two higher order factors explaining 62% of the variance. The first factor explained 45% of the variance and had an internal focus with key variables being the anxiety symptoms, ruminations and effects of anxiety experienced (evidence of pre-evaluation anxiety, experience of physiological symptoms of anxiety, negative experiences in performance, negative outcome expectancies and experience negative effects of anxiety). The second factor explained 17% of the variance and had an external focus, the worry in relation to how others viewed the individual (concern about others’ judgement). The six AMPAS variables/themes shown in Table 2 have been used (for further details see Papageorgi, 2020).

**TABLE 2:** Variables included in section C of the questionnaire.

Variable name	Example item
Negative outcome expectancies	Sometimes, before an important I find myself thinking: “This is too difficult. I am not going to do well,” even though I may have worked really hard in preparing for that event
Negative experiences in performance	During my recitals I feel great (RECODED)
Evidence of pre-evaluation anxiety	I worry a lot for several days before I take a recital examination in front of a jury
Experience of physiological symptoms of anxiety	During recitals my heart beats very fast
Concern about others’ judgement	Especially if I score low in an exam or audition, I do not tell anyone exactly what my score was
Negative effects of anxiety	I believe that anxiety makes me forget parts of the music, makes it difficult to concentrate on my playing, and eventually has a negative result on my performance

Section D included six open ended questions that required students to write down their thoughts on a number of performance-related issues that explored (1) Self-concept in music (Q: Rate yourself in relation to other young performers – scale ranging from poor to excellent. Please explain your response), (2) Perfectionistic tendencies (Q: It is important to always get top marks – scale ranging from strongly disagree to strongly agree. Please explain your response), (3) Attributions of success (Q: Remember an occasion in which you performed and did really well. Please write down what you think led to that positive result), (4) Attributions of failure (Q: Remember an occasion in which you performed but did not go as well as you hoped. Please write down what you think led to that negative result), (5) Self-awareness regarding MPA level (Q: I would describe myself as a low, moderately or highly anxious performer (choose one option) and give reasons for your answer), and (6) Strategies used to cope with performance anxiety (Q: Write down any strategies you use to help you cope with performance anxiety and perform better).

### Ethical Considerations

The research was approved by the UCL Institute of Education Research Ethics Committee (the affiliation of the author at the time the study took place). Initially, contact was made with the directors of several conservatoires and youth orchestras, and only after receiving a positive response were they approached to discuss the data collection procedure. Participation to the study was voluntary, and informed consent from parents or caregivers was obtained for all participants. Participants were briefed about the rationale and aims of the study, both orally and in writing. The questionnaire included a cover page stating the aims of the study, reassuring that all information was confidential and that participants retained the right to withdraw at any time. The researcher’s contact details were also included. Participants were assured that all information would be used for the purposes of the study and that they would remain anonymous in the dissemination of findings.

### Data Analysis

#### Quantitative Data

Quantitative data were analysed with the IBM SPSS software programme. In order to identify typologies of adolescent performing musicians, K-Means cluster analysis, an optimisation clustering technique, was used. Cluster analysis aims at grouping individuals in such a way that those that are allocated into a particular group are similar (Bartholomew et al., 2002). The aim is to discover the arrangement of observations and clusters that maximises within-group homogeneity and, at the same time, between-group heterogeneity (Everitt et al., 2011). This approach enables grouping students into clusters based on the similarity of their responses, followed by researcher-generated descriptions of these on the basis of the most prominent variables in each cluster and can therefore provide information on the presence of different student typologies (also referred to as student profiles). K-Means cluster analysis was chosen as the aim was to investigate whether distinct profiles of adolescent musicians exist on the basis of the similarities of their responses concerning MPA experienced and the strategies used to cope with the demands of performance, as the literature on test anxiety suggests for other learning domains. This technique enabled to address a yet unanswered question in the MPA literature, whether instrumental learners perceive and respond to evaluative situations in consistent but distinct ways. Data were assessed for outliers, normality, linearity and homoscedasticity of independent variables and their suitability for K-Means cluster analysis was confirmed prior to the analysis. Variables were standardized into *z*-scores to enable their comparison and to minimize any bias in weighting which may have resulted from differing measurement scales and ranges. Validation of the cluster solution was conducted through Discriminant Analysis. Associations between the emerging clusters and achievement in instrumental examinations were investigated using Pearson’s Chi-Square Test.

#### Qualitative Data

Qualitative data, derived from responses to open-ended questions, were transcribed and entered into the NVivo software programme for thematic analysis. The analysis followed an iterative process of categorisation into themes according to a seven-stage process (Cooper and McIntyre, 1993):

1. Reading a random sample of scripts.
2. Identifying points of similarity and difference among these transcripts in relation to the research questions.
3. Generating theories, on the basis of 2, describing emergent answers to the research questions.
4. Testing theories against a new set of transcripts.
5. Testing new theories against transcripts that have already been dealt with.
6. Carrying all existing theories forward to new transcripts.
7. Repeating the above process until all data have been examined and all theories tested against all data.

The coding was conducted by the author, and was later validated by two experts through a process of checking the identified themes with a random selection of scripts. If disagreements were observed, these were discussed until an agreement was reached by all parties.

The purpose of the qualitative data was to enrich the quantitative data, providing further insight into the characteristics of the student typologies. The open questions also touched upon issues not covered by the quantitative data that are important in furthering our understanding of the MPA experience for each of the typologies, such as attributions of success and failure, and coping strategies for dealing with MPA. Qualitative data facilitated further understanding of the characteristics and behaviours of each type of student in relation to the development of self-concept, perfectionistic tendencies, attributions of success and failure, self-awareness regarding performance anxiety level and strategies used to cope with performance anxiety. Four participants from each cluster group, all cases with a small distance from the classification centre of each cluster, were selected to provide further insight into the typologies of adolescent performing musicians as they were illustrative of the characteristics of each type/profile of students. Effort was made to ensure that the cases chosen were representative of the sample in terms of the key demographic variables of nationality, gender and age. The presentation of the examples is carried out according to the theme of each open ended question.

## Results

### Emerging Clusters – Typologies of Adolescent Musicians

**FIGURE 1:**
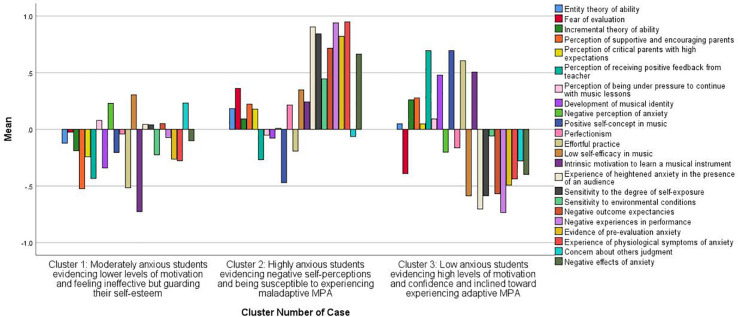
The 3-cluster solution.

The quantitative analysis initially explored various cluster solutions until the optimal was obtained. Cluster solutions of six, five, and four clusters were rejected in favour of a three cluster solution. The three cluster solution was optimal as the analysis showed that at the 9th iteration convergence was achieved due to no further change in cluster centres, signifying that the cluster model had stabilised. In the other cluster solutions iterations failed to converge at the default number of 10 iterations, suggesting that these models were not optimal. The three-cluster solution produced clusters broadly containing a similar number of students (Cluster 1: 144 students; Cluster 2: 121 students; Cluster 3: 145 students). This solution was successful in yielding three student groups with distinctively different characteristics as evidenced from the cluster centres’ mean values (Table 3 and Figure 1)^1^. One-way ANOVA with cluster membership as the independent variable confirmed the existence of statistically significant differences between the three groups in all dependent variables (*p* < 0.05) but one, which was nevertheless borderline significant (*p* = 0.05). Furthermore, the three cluster solution clearly differentiated students on the basis of reported MPA level, and grouped them into low (cluster 3), moderate (cluster 1) and high (cluster 2) MPA levels. This provided support for theoretical conceptualisations of MPA in the literature (e.g., Papageorgi et al., 2007). The effectiveness of the proposed cluster solution was further validated using discriminant analysis (see relevant section below). For all the above reasons, the 3-cluster solution was considered to be optimal. The cluster membership of each case and the distance of each case from the classification cluster centre were saved for further analysis. The final cluster centre mean values of the variables in each of the clusters were then examined to identify the most prominent variables that guided the interpretation of the clusters.

**TABLE 3:** Final cluster centre mean values for each variable included in the analysis.

	Cluster		
	1 Moderately anxious students evidencing lower levels of motivation and feeling ineffective but guarding their self-esteem	2 Highly anxious students evidencing negative self-perceptions and being susceptible to experiencing maladaptive MPA	3 Low anxious students evidencing high levels of motivation and confidence and inclined toward experiencing adaptive MPA
Entity theory of ability	−0.13	0.12	0.03
Fear of evaluation	−0.01	0.40	−0.33
Incremental theory of ability	−0.28	0.00	0.27
Perception of supportive and encouraging parents	−0.48	0.22	0.29
Perception of critical parents with high expectations	−0.27	0.20	0.10
Perception of receiving positive feedback from teacher	−0.45	−0.29	0.70
Perception of being under pressure to continue with music lessons	−0.01	−0.02	0.03
Development of musical identity	−0.34	−0.13	0.42
Negative perception of anxiety	0.17	−0.01	−0.15
Positive self-concept in music	−0.27	−0.51	0.70
Perfectionism	−0.02	0.29	−0.22
Effortful practice	−0.45	−0.27	0.66
Low self-efficacy in music	0.30	0.42	−0.64
Intrinsic motivation to learn a musical instrument	−0.65	0.14	0.52
Experience of heightened anxiety in the presence of an audience	0.02	0.87	−0.74
Sensitivity to degree of self-exposure	−0.09	0.74	−0.54
Sensitivity to environmental conditions	−0.21	0.38	−0.11
Negative outcome expectancies	0.05	0.68	−0.60
Negative experiences in performance	−0.07	0.95	−0.71
Evidence of pre-evaluation anxiety	−0.16	0.83	−0.52
Experience of physiological symptoms of anxiety	−0.27	0.90	−0.48
Concern about others’ judgement	0.25	−0.02	−0.23
Experience negative effects of anxiety	−0.14	0.66	−0.40

#### Cluster 1: Moderately Anxious Students Evidencing Lower Levels of Motivation and Feeling Ineffective but Guarding Their Self-Esteem

Cluster 1 students were strongly characterised by the fact that they reported low intrinsic motivation to learn a musical instrument. They perceived their parents not to be supportive/encouraging and they did not place great effort into practising. They also did not perceive receiving positive feedback from their teacher, suggesting that they felt inefficient and unsuccessful as performers. These students had not developed a strong sense of musical identity, had low-self-efficacy beliefs and a negative self-concept in music. They also held an entity theory of ability. Of the three groups of students, this group was the one with values closest to the mean (0) on the variables “evidence of pre-evaluation anxiety” and “experience of physiological symptoms of anxiety,” suggesting that they experienced moderate arousal levels. Qualitative data (see below) suggested that this group may have tried to protect their self-esteem through their behaviour.

#### Cluster 2: Highly Anxious Students Evidencing Negative Self-Perceptions and Being Susceptible to Experiencing Maladaptive MPA

Cluster 2 students were characterised by the fact that they had negative experiences in performance and experienced physiological arousal more intensely. They had the highest score on the variables “experience of physiological symptoms of anxiety” and “evidence of pre-evaluation anxiety.” They were more anxious in the presence of audience and experienced high levels of pre-evaluation anxiety. In addition, their anxiety was influenced by how exposed they felt when performing (they felt more anxious when playing solo as opposed to playing in an orchestra, for example), they had negative outcome expectancies, experienced the negative effects of performance anxiety, held a negative self-concept in music, low self-efficacy beliefs and perfectionistic tendencies. All the above made these students less self-confident and more vulnerable to experiencing maladaptive performance anxiety. They did not perceive receiving positive feedback from their teacher, which may suggest that they were not successful or that they tended to interpret feedback in a negative way. They also indicated that they did not invest much effort in practising, suggesting an inverse association between motivation for practice and experience of musical performance anxiety.

#### Cluster 3: Low Anxious Students Evidencing High Levels of Motivation and Confidence and Inclined Toward Experiencing Adaptive MPA

Cluster 3 students felt motivated in the presence of an audience, they had developed a positive self-concept in music and were confident players. They were intrinsically motivated, practised a lot and perceived that they received positive feedback from their teacher. They also had a strong sense of musical identity. They had some experience of physiological arousal and pre-evaluation anxiety, but their arousal and anxiety remained at low and manageable levels with values −0.52 and −0.48 on the variables “evidence of pre-evaluation anxiety” and “experience of physiological symptoms of anxiety,” respectively. They reported that they had positive experiences in performance and that anxiety had positive effects on their performance (as indicated by the minus sign on the variables “negative experiences in performance” and “comment on the negative effects of anxiety”), indicative of anxiety having a facilitative or adaptive effect.

### Validation of Cluster Solution

Discriminant analysis was carried out to explore how efficient the cluster solution was. The three clusters of students formed the groups for the discriminant analysis and the twenty-three variables used in the cluster analysis (comprising sections B and C of the questionnaire) were placed as the independent variables. Two discriminant functions were calculated, which had a significant overall Wilk’s lambda [Λ = 0.15, χ^2^ (46) = 548.64, *p* < 0.001]. After removal of the effects of the first function, the second discriminant function was also statistically significant [Λ = 0.51, χ^2^ (22) = 196.95, *p* < 0.001]. Both discriminant functions were successful at discriminating between the three groups of students. The first discriminant function accounted for 71% of the variance in the solution and the second one for 29%. The first function had an eigenvalue of 2.36 and a canonical correlation of 0.84. The η^2^, obtained by squaring the canonical correlation was 0.71, indicating that 71% of the variability of the scores for the first discriminant function was accounted for by differences among the three groups of students. The second function had an eigenvalue of 0.972 and a canonical correlation of 0.70. The η^2^ was 0.49, indicating that 49% of the variability of the scores for the second discriminant function was accounted for by differences among the three groups of students.

Function 1 was named “high anxiety and low effectiveness.” It focused on issues that made students vulnerable to experiencing maladaptive performance anxiety, such as negative experiences in performance, experience of heightened anxiety in the presence of an audience, sensitivity to the degree of self-exposure, experience of physiological symptoms of anxiety, a negative perception of oneself, low self-confidence and a lack of effort. The perception of receiving negative feedback from the teacher was also an important variable in this function.

Function 2, which was orthogonal to the first function, was named “high motivation and success.” The focus of this function was intrinsic motivation to learn a musical instrument. Other important variables included supportive and encouraging parents, effortful practice, positive feedback and development of musical identity.

The canonical discriminant functions were evaluated at group means. This allowed the three cluster groups to be viewed in relation to the two discriminant functions. Table 4 summarises the results.

**TABLE 4:** Group centroids for discriminant functions.

Cluster number of case	Function 1	Function 2
1	0.237	−1.362
2	2.018	0.851
3	−1.755	0.581

Function 1 (high anxiety and low effectiveness) mostly distinguished clusters 2 and 3. These two groups of students were at the opposite end of the spectrum with respect to performance anxiety, as cluster 3 experienced adaptive performance anxiety that enhanced performance, whilst cluster 2 experienced maladaptive performance anxiety that obstructed their performance. Function 2 (high motivation and success) mostly distinguished clusters 1 and 2. Cluster 1 included students that evidenced low levels of motivation, moderate MPA and perceptions of being less successful, whilst cluster 2 included students that were moderately motivated but experienced high MPA, and perceived themselves to be less successful as they had negative performance experiences.

Cluster group membership prediction was also assessed, to investigate how well student group membership could be predicted by using a classification function. Results suggested that 88.3% of cluster 1, 92% of cluster 2 and 97.4% of cluster 3 students were predicted correctly. Overall, 92.8% of the sample was classified correctly. In order to estimate how well the classification functions could predict a new sample, the classification was estimated by selecting the leave-one-out option. Results showed that 81.6% of cluster 1, 85.1% of cluster 2 and 94.7% of cluster 3 students were correctly classified using the leave-one-out classification. Overall, 87.5% of the cross-validated cases were correctly classified.

### Associations Between Examination Achievement and Cluster Membership

**FIGURE 2:**
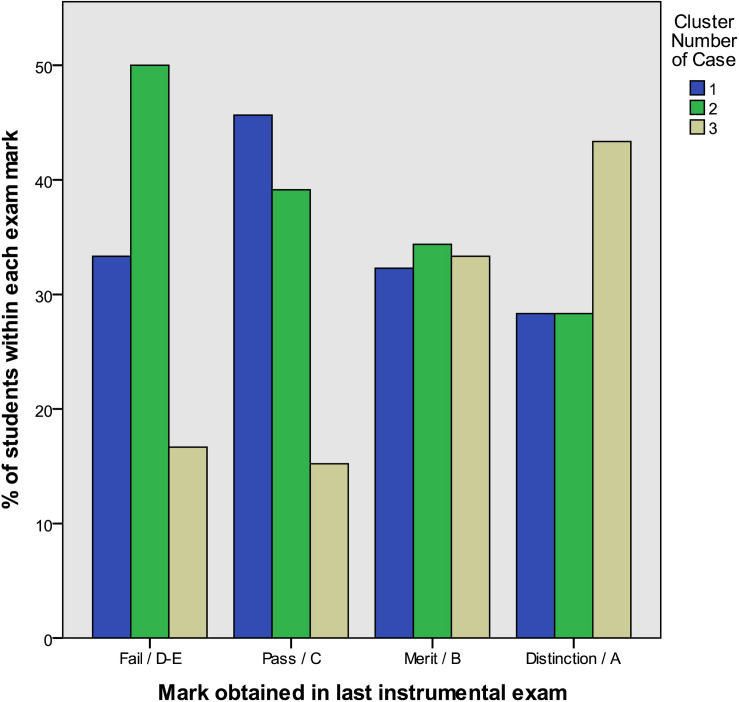
Examination achievement and cluster membership.

Pearson’s chi-square test was used to assess associations between achievement in instrumental exams and cluster membership in the data. A statistically significant association was observed between the mark obtained in the last instrumental examination students took and cluster membership [χ^2^ (6, *N* = 321) = 14.61, *p* = 0.024]. Figure 2 illustrates that cluster 1 (moderately anxious students evidencing lower levels of motivation and feeling ineffective but guarding their self-esteem) were most likely to get a Pass/Grade C, as they formed the highest percentage from the three clusters in this category. Cluster 2 (highly anxious students evidencing negative self-perceptions and being susceptible to experiencing maladaptive MPA) were most likely to either Fail or get Merit/Grade B (they formed the largest percentage of the three groups in the Fail and Merit/B categories). Cluster 3 (low anxious students evidencing high levels of motivation and confidence and inclined toward experiencing adaptive MPA) were most likely to get a Distinction/Grade A, as they formed the largest percentage from the three clusters in this category. *Post hoc* tests revealed that it was mostly marks C and A that contributed to the significant association between clusters. Significantly more Cluster 1 students and significantly less Cluster 3 students than expected received mark C (Adj. residuals = 2.1 and −3.1, respectively). Furthermore, significantly more Cluster 3 students than expected received mark A (Adj. residual = 3.0).

### Qualitative Data Representing Each Student Type

#### Cluster 1: Moderately Anxious Students Evidencing Lower Levels of Motivation and Feeling Ineffective but Guarding Their Self-Esteem

##### Development of self-concept in music

The score on the variable “perception of receiving positive feedback from teacher” (−0.45) for these students suggested that they felt the least successful of the three groups. Additionally, they had the lowest score (−0.45) on the variable “effortful practice.” Their score on the variable “positive self-concept in music” (−0.27) suggested that they held a negative self-concept. However, when asked directly about their attainment, 62.5% reported that they considered themselves to be between good and excellent performers compared to other young musicians, indicating that they held a positive perception of themselves overall. This group of students perhaps made inaccurate self-assessment in relation to performance competence or was in denial in an effort to preserve their self-esteem. They explained their responses by saying that:

*“I think I am good because I have a lot of potential and if I put my mind to it, I can achieve a lot”*

             (Male, Cypriot, 18 years old)

*“I think I am average because I am not talented, but I also don’t practise enough because I don’t have the time and so I do not perform as I well as I could. I have more potential. If I use it I believe I will reach a very high level”*

             (Female, British, 16 years old)

These responses showed that they felt that they had potential as performers but both students implied that they did not work as much as they needed to in order to realise that potential. This might indicate an effort to maintain a positive self-concept in music and to guard self-esteem by attributing an unsatisfactory performance to lack of effort rather than lack of ability. Such students protected their self-esteem by thinking that if they actually practised enough, they would be successful. This defensive mechanism may have ensured that such students’ self-esteem was not jeopardised.

##### Perfectionistic tendencies

The majority (60.4%) of students in cluster 1 agreed or strongly agreed with the statement “it is important for me to always get top marks in exams or auditions.” Some explained this by saying that this would help them achieve their goals (being accepted at university).

*“I agree because although there is more to music than exams, exams show me that I am in fact progressing well – they will stand me in good stead for uni!”*

             (Female, British, 16 years old)

Others appeared to be striving to achieve high marks because they saw it as a way of reinforcing their self-concept. High marks for this group of students might have therefore functioned as a way of ensuring they did not damage their self-esteem.

*“I agree because high marks make you feel better”*

             (Male, Cypriot, 16 years old)

##### Attributions of success

When asked to remember an occasion in which they performed well and write down what they thought led to that positive result, students in cluster 1 responded in a variety of ways and evidenced both internal and external attributions of success. On the one hand, some mentioned the importance of their effort, feeling confident and being concentrated, which indicated an internal attribution of success, but also mentioned the venue (external attribution).

*“I knew the piece very well, I was confident in my own ability to play it. Not a threatening venue*/*atmosphere – small concert, sympathetic audience. Some nerves – helped me to focus”*

             (Female, British, 18 years old)

Other students spoke about having a motive that helped them perform well and playing in a non-threatening venue/atmosphere (small audience, sympathetic audience), which pointed toward an external attribution of success. They also mentioned effort and concentration (internal attributions).

*“My effort, my concentration, the motive”*

             (Male, Cypriot, 19 years old)

It is noteworthy that some students mentioned the presence of some nerves as facilitating concentration and focus during the performance.

##### Attributions of failure

In stating reasons that led to a past unsuccessful performance, students in cluster 1 mostly provided external attributions for failure.

*“I had not felt especially nervous beforehand, so when I went on stage I suddenly felt that I was not mentally prepared. Also, more critical audience – masterclass situation”*

             (Female, British, 18 years old)

*“The atmosphere was cold and the audience was unfamiliar”*

             (Male, Cypriot, 19 years old)

*“My school teacher and I fell out and she rushed the accompaniment. I was also quite upset”*

             (Female, British, 16 years old)

These students strove to maintain a positive self-concept, and therefore attributed failures to situations beyond their control in an effort to protect their self-esteem. Some students also attributed their failure to low levels of anxiety prior to performing.

##### Self-awareness regarding performance anxiety level

The majority of students in cluster 1 described themselves as moderately anxious performers (22.7% – low anxious; 62.1% – moderately anxious; 15.2% – highly anxious). The qualitative data supported this:

*“I am anxious before the performance. When I start playing, not at all”*

             (Male, Cypriot, 18 years old)

*“I do get anxious, but it’s not uncontrollable panic”*

             (Female, British, 18 years old)

The lack of intense nerves may have been related to a lack of caring about audience perceptions, although it could also indicate further protection of self-esteem.

*“I don’t really care what other people think”*

             (Female, British, 16 years old)

##### Strategies for coping with performance anxiety

Coping strategies used by students in cluster 1 seemed to focus on maintaining a positive approach to the performance and remaining confident. They believed that when adequate preparation was made, there was no reason to get anxious.

*“Breath and just think that most people are supportive and understand the ordeal of performance”*

             (Female, British, 16 years old)

*“I think about my teacher’s advice. In order to play a piece 100%, I must know it 200%. If I have practised, there is no reason for anxiety”*

             (Male, Cypriot, 18 years old)

Maintaining a positive approach to the performance and ensuring that they practised sufficiently guarded against getting overly anxious.

Overall, students in cluster 1 appeared to be somewhat have low levels of motivation. Their comments indicated that they were driven by a desire to protect their self-concept. Some of their comments suggested one reason they might not have devoted the necessary effort in performance was in an effort to guard their self-esteem. Such behaviour allowed them to maintain a positive view of themselves despite placing low effort into practising, possibly thinking that if they had put the necessary effort they would have been successful. Reported strategies for coping with MPA were emotion-focused rather than problem-focused.

#### Cluster 2: Highly Anxious Students Evidencing Negative Self-Perceptions and Being Susceptible to Experiencing Maladaptive MPA

##### Development of self-concept in music

Almost half of the respondents in cluster 2 (48.3%) reported that they assessed themselves to be average performers and 14.2% reported to be below average or poor performers. This supported findings from the cluster analysis, which indicated negative self-concept in music and low self-efficacy beliefs in cluster 2.

*“I think I am average because I don’t believe that I have as much knowledge as I would like to in order to be a very good performer compared to others. This is mostly due to the fact that I don’t play a musical instrument”* [This student was a singer].

            (Female, Cypriot, 18 years old)

*“I think I am average because there are people who are both better and worse than me”*

             (Female, British, 18 years old)

These students seemed to construct their music self-concept by direct comparison with others. Whilst students in cluster 1 referred to their abilities and potentials and students in cluster 3 mentioned confidence and evidenced a positive approach to learning (see below), students in cluster 2 evaluated themselves on the basis of how they perceived the abilities of other young musicians.

##### Perfectionistic tendencies

Most of the students in this group (79.8%) agreed or strongly agreed that it was important to receive top marks in exams or auditions. They indicated perfectionistic tendencies, this being one of the factors that possibly triggered their maladaptive response.

*“If I do not get a high mark I feel as if I have let down myself, my teacher and my parents, as I know I am capable of achieving a high mark”*

             (male, British, 17 years old)

*“I agree that it’s important for me to get top marks in exams or auditions because others congratulate me and I feel better”*

          (Female, Cypriot, 12 years old)

Responses suggested that these students sought external approval and also felt external pressure to do well.

##### Attributions of success

Responses to questions about the factors that led to a successful performance suggested that they attributed their successes to both internal and external factors. Some mentioned being well prepared and feeling comfortable in the performance environment.

*“I was well prepared, I tried to lower my anxiety and I felt comfortable at the place I was playing”*

            (Female, Cypriot, 18 years old)

*“I was well prepared. I was playing in front of people I knew”*

             (male, British, 17 years old)

Lowering anxiety levels seemed to produce positive results for these students, and an adequate preparation and a feeling of ease in the performing environment seemed to promote this.

##### Attributions of failure

Students in cluster 2 mostly evidenced internal attributions to failure. This is an important difference from students in cluster 1, who attributed failure to external factors. Students in cluster 1 protected their self-esteem, whilst students in cluster 2, maintained and further jeopardised their low self-esteem.

*“I hadn’t practised as much as I should have, and I was very-very anxious”*

          (Female, Cypriot, 12 years old)

*“Getting too worked up about the performance, several days beforehand”*

             (male, British, 17 years old)

Most students in this group attributed their failure to the fact that they had not practised enough. It is noteworthy that these students also mentioned that they felt highly anxious and experienced pre-performance anxiety for days before the event. There was an association between lack of adequate practice and the experience of high levels of performance anxiety.

##### Self-awareness regarding performance anxiety level

Most students in cluster 2 considered themselves to be highly anxious (56.3%). In the comments they provided further details.

*“I am generally very anxious before a performance or examination due to fear of something not going well or failing the examination”*

            (Female, Cypriot, 18 years old)

*“I get very nervous generally”*

            (Female, British, 18 years old)

In justifying their response, students noted fear of failure and that they were generally anxious as individuals. The latter comment evidenced a link between trait anxiety and state anxiety, a well-documented association in MPA literature.

##### Strategies for coping with performance anxiety

Responses of students in cluster 2 suggested that they saw effective practising as a means of relieving their anxiety about performance and feeling more secure about themselves.

*“Practice – the right kind that focuses on the energy sections or especially difficult sections”*

             (Female, British, 18 years old)

They also rehearsed a lot, especially in the venue where the examination/concert was to take place.

*“Rehearsing in the exam/concert venue so that we familiarise ourselves with the environment”*

            (Female, Cypriot, 12 years old)

Student comments suggested that students in cluster 2 had developed problem-focused coping strategies. They appeared to be more proactive in their approach to coping with anxiety compared to students in cluster 1.

#### Cluster 3: Low Anxious Students Evidencing High Levels of Motivation and Confidence and Inclined Toward Experiencing Adaptive MPA

##### Development of self-concept in music

Students in cluster 3 demonstrated the most positive approach to performance and had a positive self-concept as musicians, as 90.8% of these students rated themselves between good and excellent performers. Confidence, in combination with low levels of performance anxiety, was considered to promote achievement.

*“I am good because I generally feel confident and have little anxiety in my performances”*

             (Female, British, 14 years old)

Other students mentioned that they did not feel threatened by other musicians. They considered critical listening as an opportunity to take positive elements of other musicians and saw this as a positive learning experience.

*“I am good because I want to feel confident and not get disappointed. I can take, by watching other young performers, the positive elements of their performance”.*

             (male, Cypriot, 13 years old)

##### Perfectionistic tendencies

Most of the students in cluster 3 said that getting high marks in exams or auditions was important for them (72.1%). Some saw high marks to be a reward for the hard work they put into their preparation.

*“I believe that getting high marks in exams or auditions is something like a reward for me, and so I pursue this”*

             (male, Cypriot, 15 years old)

Some students, however, disagreed with the importance given to high marks (14%). They acknowledged that an examination evaluates performance at one specific point in time, appreciated the volatility of an examination outcome due to external factors and considered that an examination result was not a reflection of ability or effort.

*“Because examinations are not a total reflection of your talent or efforts, as your playing is assessed at a time where anything good or bad can happen”*

             (Female, British, 14 years old)

Overall, students in cluster 3 saw achievement as a reward for their hard work, but at the same time held a more pragmatic view, acknowledging that one specific examination result could not be a valid reflection of capability and effort.

##### Attributions of success

Students in cluster 3 attributed successful past performances both to internal and external factors. For instance, effort and teacher support.

*“The effective and systematic practice and the support I received from my teacher”*

             (male, Cypriot, 13 years old)

Some students referred to their commitment to the music.

*“I felt confident and I really loved the piece and I probably conveyed that to the judges and audience”*

             (Female, British, 14 years old)

##### Attributions of failure

Failure was attributed to both internal and external parameters by students in cluster 3. Some students attributed failure to anxiety because of lack of effort.

*“Unfortunately, on some occasions I did not manage to give my best because of anxiety. I felt that I hadn’t practised the pieces as much as I should have and that made me give an anxious performance”*

             (male, Cypriot, 15 years old)

Other factors relating to organisation issues and the venue were also mentioned.

*“Poor organisation on competition’s part, unsatisfying venue, leading to awkward feelings about playing”*

             (Female, British, 15 years old)

##### Self-awareness regarding performance anxiety level

Most students in cluster 3 perceived themselves to be moderately anxious performers (51.8%), although a significant percentage rated themselves as low anxious (39.7%). Students in cluster 3 experienced lower levels of physiological arousal compared to the other two clusters (see Table 3). Students raised a number of points in explaining their responses, such as enjoying performing rather than feeling threatened by it.

*“I see music as something that pleases me and not as something that makes me anxious”*

             (male, Cypriot, 15 years old)

Other students spoke about being able to use performance anxiety to enhance the quality of their performance.

*“Though I am usually quite anxious about performing, I usually manage to use this to my advantage to play well”*

             (Female, British, 15 years old)

##### Strategies for coping with performance anxiety

Coping strategies used by students in cluster 3 focused on having a positive attitude to the performance.

*“I just think positively”*

             (male, Cypriot, 15 years old)

Other students also mentioned focusing on the positive things of their performance as opposed to focusing on the things that they did not like, as well as concentrating on communicating with the audience through their performance.

*“Have a really positive train of thought, think of the good things that you do in the piece and try to love the piece as much as possible because it will show in your performance and therefore will have a positive effect on the audience”*

             (Female, British, 15 years old)

These students had developed active problem-focused strategies for coping with anxiety, not only before the performance, but also during the actual performance.

## Discussion

The cluster analysis revealed the existence of three typologies or profiles of instrumental students: “moderately anxious students evidencing lower levels of motivation and feeling ineffective but guarding their self-esteem,” “highly anxious students evidencing negative self-perceptions and being susceptible to experiencing maladaptive MPA,” and “low anxious students evidencing high levels of motivation and confidence and inclined toward experiencing adaptive MPA.” An inspection of both quantitative and qualitative data assisted in a more thorough understanding of the characteristics of each of these student types and their relationship with the experience of performance anxiety.

Cluster 1, moderately anxious students evidencing lower levels of motivation and feeling ineffective but guarding their self-esteem, were characterised by the fact that they reported low intrinsic motivation to engage in instrumental learning, placed low effort into practising and perceived their parents not to be supportive. They had a weak sense of musical identity and were troubled by low self-efficacy and negative self-concept beliefs. These students experienced moderate arousal levels and felt that they were not successful (they reported that the feedback they received from their teacher was negative). Inspection of the qualitative data suggested that these students strove to guard their self-esteem. High marks for these students served to reinforce self-concept. They attributed their failures to external factors that were beyond their personal control, a behaviour that might have served to protect self-esteem. With respect to their attitudes regarding achievement, there was indication that most had perfectionistic tendencies, and some saw success as a way of reinforcing their self-concept (“feeling better”). This group of students employed emotion-focused rather than problem-focused strategies. Most students focused on ensuring they felt positive and confident before the performance, something that they felt would guard them against feeling too anxious. These students were most likely to do moderately well (get a Pass) in instrumental examinations, which indicated that they did well enough to make sure they did not jeopardise their self-esteem. The coping strategies used, in combination with the attempt to guard self-esteem and lower levels of motivation correspond with some of the characteristics of cluster 1 (Tests out of control) in Davis et al. (2008).

Cluster 2, highly anxious students evidencing negative self-perceptions and being susceptible to experiencing maladaptive MPA, were characterised by the fact that they had negative experiences in performance, experienced physiological arousal more intensely and held negative views about themselves and their ability as performers. They were often apprehensive about an upcoming performance days before an event and usually had negative outcome expectancies. They commented strongly on the negative effects that anxiety may have on performance. Their personal characteristics included negative self-concept in music, low self-efficacy beliefs and perfectionism. They perceived the feedback that they received from their teacher to be negative and invested little effort in practice. This finding, in conjunction with the frequent negative outcome expectancies that they reported, supports earlier research which suggests that estimations of high probability of failure tend to result in the investment of little effort (Covington and Omelich, 1981; Carver and Scheier, 1986). Qualitative data suggested that these students tended to compare themselves with others and generally held negative self-views. Moreover, they felt the need for external approval and pressure to achieve in music, and perhaps this was the reason they considered achieving high marks to be so important. They tended to attribute their failures to internal factors, which may explain their negative self-concepts and low self-efficacy beliefs and perhaps contributed to maintaining their low self-esteem and promoted sensitivity to maladaptive performance anxiety. Weiner (1985) stressed the importance of achievement attributions, suggesting that when negative events are explained in terms of internal, stable, and controlled factors, students feel more pessimistic. They were self-aware regarding how anxious they were as performers, which may explain the use of proactive and problem-focused strategies for coping with performance anxiety, such as practising adequately and rehearsing in the performance venue in advance of an examination/concert. The importance of acclimatisation within the performance environment when students prepare for a performance has been highlighted by Hallam (2006), and is a view reinforced by the findings of the present study. These students were most likely to either Fail or do well (get Merit) in instrumental examinations. This might imply that when students were not in control of their nerves, these impeded their performance, whilst when they were able to cope with them they were able to perform well. This group had some similar characteristics to those described in cluster 3 (Feeling hopeless) by Davis et al. (2008) in terms of experiencing higher anxiety levels and good achievement (grade B). Nevertheless, in the current study some participants in this cluster of highly anxious students noted that they Failed in examinations, a finding consistent with cluster 1 (high anxiety/low academic buoyancy) in Putwain and Daly (2013) who had the lowest academic achievement. This latter finding supports literature linking physiological arousal with performance efficiency (Yerkes and Dodson, 1908; Wilson, 2002; Papageorgi et al., 2007), relating the experience of high levels of physiological arousal with maladaptive performance anxiety and the impairment of performance skills.

Cluster 3, low anxious students evidencing high levels of motivation and confidence and inclined toward experiencing adaptive MPA, were characterised by positive experiences in performances. When compared to the other two groups, they experienced lower arousal and anxiety levels in performance. Students in this group were generally confident, had a strong sense of musical identity, a positive self-concept in music and enjoyed their engagement with musical activities, without feeling threatened by the presence of an audience. They were intrinsically motivated and reported that they devoted a lot of time to practising. They perceived the feedback that they received to be positive. Although their physiological arousal levels were not at zero level (which would impede concentration on the task), these were maintained at a low and manageable level that eventually helped them perform well. The qualitative data reinforced these findings, showing that these students perceived themselves to be highly competent. They held a more pragmatic view on the importance of achievement. Achieving high marks in examinations and auditions was important for them, not because they sought external reassurance of their abilities, but because they saw this as a reward for their hard effort. They maintained a healthy attitude to such rewards, as they acknowledged that an examination result represented the quality of their performance at a specific point in time, and not their general ability in music. These students’ attitudes in attributing failure in performance were a balanced combination of internal and external factors. They were self-aware in relation to their anxiety levels and stressed that they did not feel threatened by performance and that they engaged in this activity because it was something they inherently enjoyed doing. They were able to use the arousal they felt prior to performance in a positive way and believed that this enhanced the quality of their playing, supporting research stating that performance anxiety can lead to positive outcomes under certain conditions (Hamann, 1982; Gates and Montalbo, 1987; Wills and Cooper, 1988; Kemp, 1996; Papageorgi, 2008; Papageorgi et al., 2013). Overall, these students maintained a healthy, balanced and thoughtful approach to performance, which was also evident in the coping strategies they employed. They emphasised the importance of maintaining a positive approach to performance in dealing with performance anxiety (emotion-focused strategies) but also employed problem-focused strategies that were activated during performance, e.g., focusing on the music and communication. These students were most likely to do very well (get Distinction) in instrumental examinations, which further reinforced the fact that any anxiety experienced did not impede their performance but helped them perform at their best. The characteristics of this group are in agreement with cluster 2 (well-prepared for challenges) in Davis et al. (2008) and cluster 2 (low anxiety/high academic buoyancy) in Putwain and Daly (2013), where students with low anxiety levels evidenced high levels of achievement and higher levels of academic buoyancy. Evidence for cluster 3 suggest the development of resilience and successful coping with MPA, resulting in pre-performance arousal facilitating performance rather than hindering it.

Differences between the three clusters of students were observed in relation to the strategies they employed for coping with performance anxiety. Moderately anxious students evidencing lower levels of motivation and feeling ineffective but guarding their self-esteem (cluster 1) did not report any strategies other than being adequately prepared in order to minimise their anxiety, indicating that they were not proactive in developing problem-focused strategies specifically for performance, but their approach was emotion-focused. Highly anxious students evidencing negative self-perceptions and being susceptible to experiencing maladaptive MPA (cluster 2) mentioned effective practising, but also mentioned proactive problem-focused strategies, such as rehearsing in the venue prior to the performance. This indicates that these students planned their performance, perhaps in an effort to minimise the negative effects of anxiety. Whilst cluster 1 and 2 students’ strategies mostly focused on strategies they could employ themselves, low anxious students evidencing high levels of motivation and confidence and inclined toward experiencing adaptive MPA (cluster 3) were able to shift their attention away from themselves. They mentioned concentrating on the enjoyment of music and focusing on communication with the audience in addition to maintaining a positive approach prior to performance. This indicated that cluster 3 students had devised active problem-focused coping strategies and that their thinking about performance had progressed to a more advanced level.

Overall, student responses are suggestive of differences in the way that each cluster approached instrumental learning and performance, as well as their experiences of MPA. Several of the concepts included in the study relate to concepts of motivation theory. Several findings from this study can be interpreted through the lens of the expectancy-value theory of achievement motivation, which states that individuals’ choice, persistence, and performance on an activity can be explained by their expectations of success (beliefs about how well they will perform) and its incentive value [the extent to which they value the activity (Wigfield and Eccles, 2000)]. For example, highly anxious students’ negative perceptions of their ability (on the basis of negative experiences in performance and negative feedback received from significant others) in combination with negative self-perceptions (low self-efficacy and low self-concept in music) and perfectionistic tendencies lead to negative outcome expectancies and investment of little effort in practice (low motivation for practice) in high-stakes situations (such as examinations). These conditions can increase MPA levels, resulting in additional negative experiences in performance and, in the long-term, they can contribute to more negative self-perceptions that further increase susceptibility to maladaptive anxiety in the future.

Current literature suggests that adolescence is a time of increased vulnerability to MPA (Fehm and Schmidt, 2006; Kenny and Osborne, 2006), and recent studies have revealed the presence of a developmental trajectory of MPA, with performance anxiety appearing to increase from childhood to adolescence and then throughout adolescent years (Osborne et al., 2005; Patston and Osborne, 2016; Papageorgi, 2020). Professional experience could act as a protective factor, as literature has suggested that the impact of anxiety on performance is mediated by musicians’ performance experience when comparing higher education students with professional musicians (Papageorgi et al., 2013). It is important to note at this point that we must not ignore the possibility that some younger or still developing musicians suffering severely from MPA may have ceased their engagement with instrumental learning and so the adolescent, adult and professional musicians’ samples presented in the literature may not include those who have experienced extreme MPA levels and severe negative effects as children.

## Limitations

Cluster analysis is a descriptive technique, which can result in solutions that are non-unique (Romesburg, 1984). A three-cluster solution was chosen as it was effective in distinguishing between different types of students and was supported by theoretical conceptualisations of MPA in the literature, but it should be acknowledged that the interpretation encompasses some degree of subjectivity. Although the cluster solution was validated through discriminant analysis, which provided support for the chosen interpretation, other researchers may have theoretical or statistical reasons to disagree. Another limitation is the use of self-report instruments, which may increase the likelihood of response bias (Bradburn et al., 2004). Future research could investigate the extent to which the three-cluster solution is replicated in other samples of instrumental musicians, not only children and adolescents but also adults. As the data were collected via a self-report questionnaire, assumptions about causality cannot be made. This study could, however, serve as a starting point for further research looking into cause-and-effect relationships between the variables that have been identified as being associated with the experience of MPA.

## Conclusion

This study adds to the body of MPA literature by exploring the different ways with which adolescent musicians interpret and respond to anxiety inducing situations. Findings have implications for clinical and educational practice. For example, interventions for students reporting low levels of motivation who feel less successful but, through their behaviours, aim to guard their self-esteem should focus on improving their sense of self and sense of achievement and reinforce that a person’s self-worth should not depend on achievement grades. For students who experience high levels of performance anxiety, interventions should concentrate on techniques for managing physiological arousal and reducing the negative effects on the quality of performance. The aim should be to facilitate the experience of success to support the development of a positive sense of self and increase intrinsic motivation. Better understanding of how MPA is manifested and the different ways with which individuals experience and respond to evaluative situations is critical in the design of tailor-made intervention programs aiming at reducing MPA in adolescents, as well as in planning appropriate educational support. The ultimate aim should be to facilitate positive performance experiences so that students derive pleasure and benefit from their engagement with music.

## Data Availability Statement

The raw data supporting the conclusions of this article will be made available by the authors, without undue reservation.

## Ethics Statement

The studies involving human participants were reviewed and approved by the UCL Institute of Education Research Ethics Committee. Written informed consent to participate in this study was provided by the participants’ legal guardian/next of kin.

## Author Contributions

IP conceptualised the study, collected and analysed the data, and authored the manuscript.

## Conflict of Interest

The author declares that the research was conducted in the absence of any commercial or financial relationships that could be construed as a potential conflict of interest.

## Footnotes

1 Variables are standardised, with a mean of 0 and a standard deviation of 1.
